# Developing Endemicity of Schistosomiasis, Corsica, France

**DOI:** 10.3201/eid2701.204391

**Published:** 2021-01

**Authors:** Camilla Rothe, Thorbjörn Zimmer, Mirjam Schunk, Claudia Wallrauch, Kerstin Helfrich, Fatih Gültekin, Gisela Bretzel, Jean-François Allienne, Jérôme Boissier

**Affiliations:** University Hospital, LMU Munich, Munich, Germany (C. Rothe, T. Zimmer, M. Schunk, C. Wallrauch, K. Helfrich, F. Gültekin, G. Bretzel);; Université de Perpignan Via Domitia, Université de Montpellier, Perpignan, France (J.-F. Allienne, J. Boissier)

**Keywords:** Schistosoma, *S. hematobium*, *S. bovis*, bilharzia, neglected tropical diseases, hematuria, travel medicine, parasites, Corsica, France, urinary blood fluke, schistosomiasis

## Abstract

Urogenital schistosomiasis was diagnosed in a man from Germany who had never traveled outside Europe. He likely acquired the infection in Corsica, France, but did not swim in the Cavu River, which was linked to a previous outbreak. This case highlights that transmission of schistosomiasis in Corsica is ongoing.

A 49-year-old man from Germany experienced macrohematuria in June 2020 and underwent cystoscopy in August 2020. Histologic analysis of a bladder biopsy specimen showed ova of *Schistosoma* spp. He was referred to the outpatient department for tropical medicine at LMU Hospital Munich.

The patient had never traveled outside Europe. He had, however, traveled twice to Corsica, France, in 2013 and 2019. He never swam in the Cavu River, which has been associated with cases of schistosomiasis in recent years (*1*–*3*).

The patient visited Corsica during August 22–September 4, 2019. By using GPS data from his smartphone and camera, he reconstructed his bathing sites precisely. During August 22–24, he swam several times in the Solenzara River in the southeastern part of the island, near a busy campsite (Figure). Further minor water contacts might have occurred at the Gravona River in western Corsica near Ajaccio, at a turtle park and near a campsite, and at the Tavignano River (Figure). He did not bathe in either of Corsica’s other rivers but could not rule out contact with water for cooling purposes. The patient reported swimming in the Restonica River in 2013 (Figure), but he did not recall any itchy rash suggestive of cercarial dermatitis.

**Figure:**
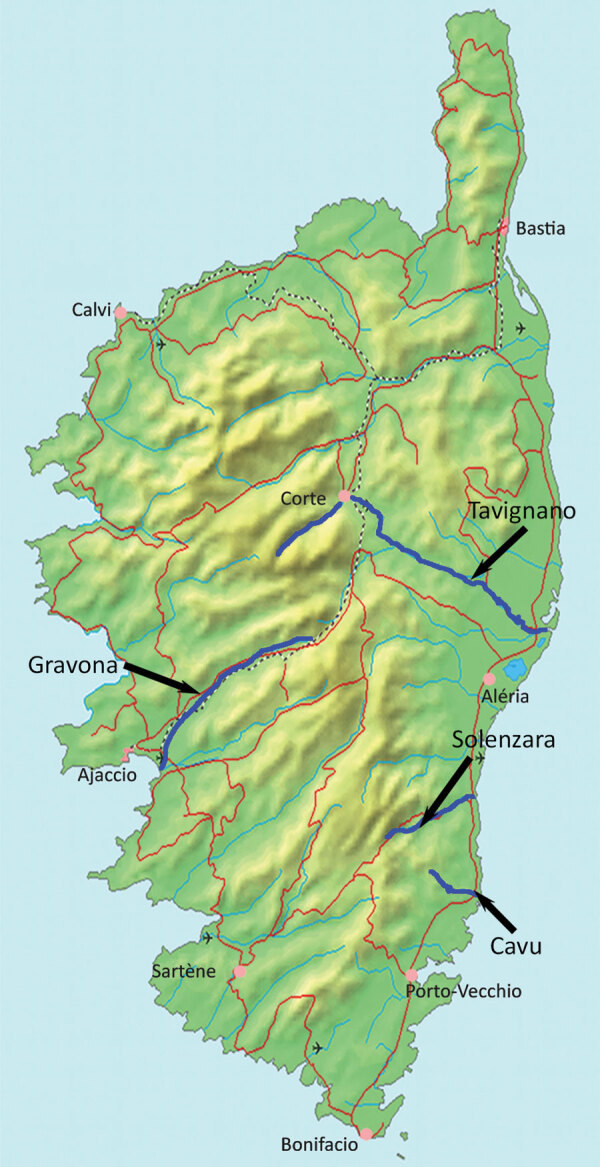
Locations of rivers referenced in investigation of developing endemicity of schistosomiasis, Corsica, France. Map adapted by using UBehrje (https://www.demis.nl); in public domain (https://upload.wikimedia.org/wikipedia/commons/3/3e/Corsica_Map.png).

Physical examination was unremarkable. Full blood count did not show any eosinophilia. Urine sediment microscopy showed terminal-spined ova resembling those of *S. hematobium* parasites. Urine tested in-house by quantitative PCR for *S. hematobium* parasites was positive. A schistosoma serologic test was positive by enzyme immunoassay (64 U/L; reference <10 U/L); results of a blood immunochromatographic rapid diagnostic test were positive, but Western blot results were negative. Results of a urine circulating cathodic antigen point-of-care test was weakly positive. His son, who traveled with him in 2013, and his wife, who had joined him on both trips, had negative serologic test results and were negative for urine schistosoma by PCR.

DNA extracted from 7 ova was successfully amplified on nuclear internal transcribed spacer and mitochondrial cytochrome oxidase I markers. For the nuclear marker, DNA (995 bp) of all eggs showed a typical signature of *S. hematobium* parasite. The mitochondrial marker (873 bp) of all eggs had a typical signature of *S. bovis* parasite and displayed the same haplotype. In summary, all eggs were identified as hybrids of *S. hematobium* and *S. bovis* (ShxSb) parasites. This type of hybrid (nuclear Sh and mitochondrial Sb) was the most frequently observed type during the 2013 outbreak (*4*). The mitochondrial cytochrome oxidase I sequences of all eggs had 100% homology with haplotype Sb2, the most frequent haplotype observed during the 2013 outbreak (*4*). Presence of this haplotype was also documented in subsequent reports on the first outbreak in Corsica (*2**,**3*).

Transmission of schistosomiasis in Corsica has been documented since 2013 (*3*,*5*). Cases have been detected in residents and in tourists (*5*,*6*). In spring 2014, after the first outbreak, intense screening and public health efforts led to the identification of 106 cases linked to the Cavu River (*7*). During 2015–2018, sporadic cases were detected and linked to the Cavu (*8*). The cases reported in 2018 involved 2 patients who had bathed in the Cavu and Solenzara Rivers (*3*).

The patient we describe reported having no contact with the Cavu River. Instead, the infection was most likely acquired in the Solenzara River, near a busy campsite. The Solenzara neighbors the Cavu, but their waters do not intermingle. No confirmed cases acquired in the Solenzara River have been documented, but *Bulinus truncatus* snails or their DNA have been found along the river during environmental surveys (*1*,*9*). Snail density close to the main bathing site of our patient appears to be high (*9*). Neither of the other rivers mentioned was ever proven to be a source of schistosomiasis; in addition, they are all relatively far away from the Cavu region.

Genotyping revealed the *S. hematobium*–*S.bovis* hybrid parasite described in previous outbreaks, suggesting ongoing transmission rather than reintroduction. The parasite’s emergence in another river cannot be explained by the persistence of infected snails (*9*) but could be explained by reseeding of the river by a mammalian host.

Animal reservoirs have been discussed as a possible explanation for ongoing transmission; however, evidence of a major role is lacking. No infection has been detected in livestock in the region, and the only infected animals found were 2 rats (*10*). Even if we cannot rule out the influence of an undetected animal reservoir (e.g., *Ovis aries musimon,* wild sheep native to Corsica, have never been tested), the most likely explanation is that 1 or several infected persons continue to infest the water.

In summary, this case highlights that transmission of schistosomiasis in Corsica is ongoing and is no longer restricted to the Cavu River. The parasite appears to be of the same strain detected previously on the island. The infection was acquired at a frequented tourist site, suggesting that more persons might have been infected. Further screening of residents and tourists is urgently needed.
